# Peripheral Blood Neutrophils and Monocytes Predict Disease Progression in Stage III Melanoma Patients Treated With Anti Programmed Cell Death Protein‐1 (PD‐1) Inhibitors

**DOI:** 10.1155/jimr/3825424

**Published:** 2026-03-22

**Authors:** Annagioia Ventrici, Luca Modestino, Leonardo Cristinziano, Francesco Caraglia, Ilaria Spatocco, Carlo Gabriele Tocchetti, Teresa Troiani, Remo Poto, Paola de Candia, Gilda Varricchi, Maria Rosaria Galdiero

**Affiliations:** ^1^ Department of Translational Medical Sciences (DiSMeT), University of Naples “Federico II”, Naples, Italy, unina.it; ^2^ Department of Internal Medicine and Clinical Immunology, University Hospital of Naples “Federico II”, Naples, Italy, unina.it; ^3^ Center for Basic and Clinical Immunology Research (CISI), University of Naples “Federico II”, Naples, Italy, unina.it; ^4^ Oncoematology Unit, Department of Precision Medicine, University of Campania “Luigi Vanvitelli”, Naples, Italy, unina2.it; ^5^ Department of Molecular Medicine and Medical Biotechnology, University of Naples “Federico II”, Naples, 80131, Italy, unina.it

**Keywords:** checkpoint inhibitors, innate immunity, melanoma, monocytes, neutrophils

## Abstract

Melanoma is a global health issue, with an increasing incidence in recent years. Immune checkpoint inhibitors (ICIs, e.g., antiprogrammed cell death protein 1 [PD‐1]) and targeted therapies (e.g., BRAF/MEK inhibitors) have revolutionized treatment for melanoma patients (MPs), but prognostic markers of drug response and disease progression remain elusive. We conducted a prospective baseline and follow‐up study to investigate peripheral blood neutrophils and monocytes as immune biomarkers in stage III MPs receiving either anti‐PD‐1 therapy or B‐type rapidly accelerated fibrosarcoma (BRAF)/mitogen‐activated protein kinase kinase (MEK) inhibitors. Sixty‐four stage III MPs were prospectively recruited, of whom 42 received anti‐PD‐1 therapy, and 22 received BRAF/MEK inhibitors. Neutrophils and monocytes were isolated and analyzed by flow cytometry, while plasma concentrations of neutrophil‐related mediators and neutrophil extracellular trap (NET) biomarkers were measured by enzyme‐linked immunosorbent assay (ELISA). In MPs, neutrophils displayed an activated phenotype (CD16^+^ CD62L^−^), and both neutrophils and monocytes had higher PD‐1 ligand (PD‐L1) expression, compared with healthy controls (HCs). Importantly, higher percentages of CD16^+^ CD62L^–^ and PD‐L1^+^ neutrophils and PD‐L1^+^ monocytes—but not higher levels of neutrophil‐related mediators and NET biomarkers—were associated with disease progression only in MPs treated with anti‐PD‐1 therapy but not in those treated with BRAF/MEK inhibitors. This study reveals that a specific neutrophil and monocyte phenotype can predict clinical responses exclusively in ICI‐treated patients, highlighting their potential as prognostic biomarkers before starting the immune checkpoint inhibition in melanoma.

**Summary**

• Elevated frequencies of CD16^+^ CD62L^–^ and PD‐L1^+^ neutrophils and PD‐L1^+^ monocytes are associated with disease progression in stage III MPs receiving anti‐PD‐1 therapy.

## 1. Introduction

Melanoma has become a major global public health concern in recent decades. The incidence of cutaneous melanoma is increasing worldwide, and several studies indicate that it has doubled in the past 10 years [1, 2]. Skin melanoma is the second most common type of cancer in men and the fourth most common type of cancer in women [3]. Indeed, males are 1.5 times more likely than females to develop melanoma [3]. The 5‐year overall survival (OS) rate for patients with metastatic melanoma has been less than 10% until recently, indicating a dismal prognosis. The advent of new therapeutic approaches, including immunotherapy (IT) with immune checkpoint inhibitors (ICIs) (e.g., anticytotoxic T‐lymphocyte‐associated protein 4 [CTLA‐4] and antiprogrammed cell death protein 1 [PD‐1]) and/or targeted therapy (TT) with B‐type rapidly accelerated fibrosarcoma (BRAF)/mitogen‐activated protein kinase kinase (MEK) inhibitors, has improved these patients’ chances of survival in recent years [4, 5].

Tumor IT has achieved a new milestone with anti‐PD‐1 monoclonal antibodies, as ICI therapy targeting the PD‐1/PD‐1 ligand (PD‐L1) axis has shown promising results in various malignancies in recent years [6], including melanoma [7]. Indeed, cancer cells can express PD‐L1, which inhibits the cytotoxic function of immune cells, leading to immune evasion, uncontrolled proliferation, and tumor progression [7]. Binding PD‐1 on T cells inhibits T cell receptor (TCR) activation and the costimulation signal transduction [8]. This mechanism suppresses T cell activation and proliferation, reduces effector T cell function, and allows tumor cells to bypass the immune system.

With regards to TT, the discovery of activating *BRAF* mutations in human tumors has underlined the importance of the microtubule‐associated protein (MAP) kinase pathway, which includes B‐Raf, MEK1/2, and ERK1/2, for tumor cell proliferation and survival. B‐Raf kinase inhibitors retain therapeutic potential in cancers bearing *BRAF* oncogene mutations, as well as in cancers bearing activating mutations of Ras and/or growth factor receptors [9]. The activated receptor tyrosine kinase induces guanosine triphosphate (GTP)–binding to Ras upon growth factor binding. B‐Raf is drawn to the membrane by GTP‐bound Ras, triggering the Raf/MEK/ERK kinase cascade. Mutations in *RAS* or *BRAF* can constitutively activate this pathway [9]. In fact, all melanoma cell lines have high MAP kinase activity, and small molecule inhibitors of the downstream kinase MEK, such as dabrafenib and trametinib, can prevent their proliferation [10]. Compared with dabrafenib monotherapy, the combination of dabrafenib and trametinib resulted in notable improvements in progression‐free survival (PFS) and OS rates in patients with stage IIIc or IV melanoma [11]. Additionally, the combination group demonstrated a greater overall response rate (ORR) (67%) than the dabrafenib alone group (51%) [12]. Relapses within the first 12 months of therapy are more likely in patients treated with adjuvant IT than in those administered adjuvant BRAF‐TT [13–15]. Thus, there is considerable interest in discovering potential biomarkers for determining which patient subgroups will benefit from these therapies.

Growing evidence shows that innate immunity plays a critical role in cancers, such as melanoma, which is highly immunogenic because of its high mutational burden [16, 17]. There is increasing evidence that polymorphonuclear neutrophils (PMNs) are of critical importance among the innate immune cells [18, 19]. PMNs are characterized by extensive plasticity [20], and several triggers in the tumor microenvironment (TME) can modify their functions toward protumor or antitumor phenotypes [21]. PMNs in the TME and peritumoral tissue express a high level of PD‐L1 [22, 23]. PMNs can colocalize and actively interact with T cells [19, 24]. Indeed, PD‐L1^+^ PMNs can directly inhibit T cell responses and promote tumor growth [25]. Moreover, activated PMNs release neutrophil extracellular traps (NETs), an extracellular fibrillary network composed of nuclear components (DNA and histones) and granule proteins [26]. NETs play key roles in various diseases, including cancer [27, 28]. Monocytes also play a role in metastatic melanoma; patients with elevated monocyte counts in the peripheral blood have a decreased chance of survival [29].

We recently established that the frequency of peripheral blood PD‐L1^+^ PMN predicts disease progression and response to anti‐PD‐1 therapy in patients with stage IV BRAF wild‐type melanoma [30]. We also reported that conditioned media (CMs) derived from melanoma cell lines produce soluble mediators able to “educate” PMNs toward an activated functional state [31]. To our knowledge, the possible role of PMNs and monocytes as disease and therapy biomarkers in patients with stage III melanoma treated with IT and/or TT has not yet been well defined. In this study, we conducted a baseline and follow‐up evaluation of peripheral blood PMNs and monocytes from melanoma patients (MPs) who received anti‐PD‐1 therapy and/or BRAF/MEK inhibitors.

## 2. Materials and Methods

### 2.1. Patients, Treatment, and Assessment

We performed an observational cohort study by prospectively recruiting stage III MPs who were candidates for PD‐1 inhibitor (nivolumab) or BRAF/MEK inhibitor (i.e., dabrafenib and trametinib) treatment after complete resection of the primary lesion and single lymph node biopsy (SLNB) or complete lymph node dissection (CLND) according to the clinical features of the patients’ lymph nodes. A total of 64 MPs had a diagnosis of stage III melanoma, according to the VIII edition of the American Joint Committee on Cancer [32], at the Oncoematology Unit, Department of Precision Medicine, University of Campania “Luigi Vanvitelli,” Naples. After surgery, 42 out of 64 MPs were treated with anti‐PD‐1 therapy, and 22 were treated with BRAF/MEK inhibitors. The patients’ characteristics, including sex, age, distant metastasis status, *BRAF* mutation status, and line of therapy, are summarized in Table 1. Patients were treated with TT or IT based on the presence of a BRAF targetable mutation (V600) and their compliance with the two modalities. For TT, 300 mg dabrafenib + 2 mg trametinib was given orally every day for 1 year. In the IT arm, nivolumab (240 mg q2w or 480 mg q4w) was administered intravenously for 1 year. For the latter, comorbidities, patient age, and compliance were the criteria considered for the investigator’s choice. The response to therapy was evaluated according to the Response Evaluation Criteria In Solid Tumors (RECIST) V.1.1 criteria [33]. Radiological (magnetic resonance imaging [MRI] or computed tomography [CT] scans of the brain, bone, chest, abdomen, pelvis, and other soft tissue as applicable) and visual (skin lesion) tumor assessments were performed at baseline and every 12 weeks until progression or the discontinuation of therapy per RECIST. According to these criteria, “no evidence of disease” was defined as the absence of clinical or radiographic evidence of disease recurrence documented within 6 months of the start of therapy. All patients provided written informed consent for the use of samples in accordance with institutional regulations. All patients’ peripheral blood samples were obtained and processed fresh at baseline (before beginning therapy, on the first cycle day) and every 12 weeks thereafter. Moreover, blood samples from 55 sex‐ and age‐matched healthy controls (HCs) were collected at the Center for Basic and Clinical Immunology Research (CISI) of the University of Naples Federico II, Naples, Italy. Plasma samples were obtained (400 g and +4°C) and stored at −80°C until use. The study was approved by the local Ethics Committee of the University of Campania Luigi Vanvitelli, Naples (n. 59), and University of Naples Federico II (n. 301/18). The study was conducted in accordance with the provisions of the Declaration of Helsinki.

**Table 1:** Demographic and clinical characteristics of melanoma patients (MPs) and healthy controls (HCs) at baseline.

Demographic and clinical characteristics	Melanoma patients		Healthy controls	
	*N*	%	*N*	%
**Age**				
Median, years**^a^**	59	—	58	—
Range	21–83	—	26–75	—

**Gender**				
Male	44	68.8	35	54.7
Female	20	31.3	20	31.3

***BRAF* mutation**				
No	24	37.5	NA	NA
Yes	40	62.5	NA	NA

**Lymph node involvement**				
N0	5	7.8	NA	NA
N1	41	64.1	NA	NA
N2	5	7.8	NA	NA
N3	2	3.1	NA	NA

**Therapy**				
Nivolumab	42	65.6	NA	NA
Dabrefenib/trametinib	22	34.4	NA	NA

**Best response to therapies**				
Nivolumab	42	—	—	—
PD	20	47.6	NA	NA
NED	22	52.4	NA	NA
Dabrefenib/trametinib	22	—	—	—
PD	9	40.9	NA	NA
NED	13	59.1	NA	NA

Abbreviations: *N*, number; NA, not available; NED, no evidence of disease; PD, progressive disease.

**^a^** Age entered as continuous variable.

### 2.2. Flow Cytometry Analysis

MP and HC blood samples (20 mL) were collected into ethylenediaminetetraacetic acid (EDTA) vacutainers (Becton Dickinson, NJ, USA), and peripheral blood mononuclear cells (PBMCs) and PMNs were isolated within 2 h of blood collection. Leukocytes were separated from erythrocytes by 3% dextran sedimentation (PanReac AppliChem ITW Reagents, Darmstadt, Germany). PBMCs and PMNs were separated via Ficoll‒Paque Histopaque‐1077 (Sigma‒Aldrich, Darmstadt, Germany) density gradient centrifugation (400 ×*g*, 20 min, and +22°C). PMNs obtained after Ficoll‒Paque were washed in phosphate‐buffered saline (PBS) and purified via Percoll (65%) (Sigma‒Aldrich, Darmstadt, Germany) density gradient centrifugation (660 ×*g*, 20 min, and +22°C), as previously described [34]. PBMCs and PMNs were incubated in Roswell Park Memorial Institute (RPMI) with 10% fetal bovine serum (FBS) and antibiotics for 30 min at +37°C and then washed with PBS. Then, 5 × 10^5^ cells per well were seeded in U‐shaped 96‐well plates, and Zombie Violet dye (BioLegend, San Diego, CA, USA) was added to evaluate cell viability (20 min and +4°C). PBMCs were stained (20 min, +4°C) in PBS containing 1% FBS with the following antibodies: fluorescein isothiocyanate (FITC)–conjugated anti‐CD14 (1:50, from Miltenyi Biotec, Bergisch Gladbach, Germany) and phycoerythrin (PE)–conjugated anti‐PD‐L1 (1:10, from Biolegend, CA, USA). Moreover, PMNs were stained (20 min and +4°C) in PBS containing 1% FBS with the following antibodies: allophycocyanin (APC)–conjugated anti‐CD66b (1:50, from Miltenyi Biotec, Bergisch Gladbach, Germany), peridinin chlorophyll protein (PerCP)–conjugated anti‐CD11b (1:50, from Miltenyi Biotec, Bergisch Gladbach, Germany), VioBlue‐conjugated anti‐CD193 (1:10, from Miltenyi Biotec, Bergisch Gladbach, Germany), FITC‐conjugated anti‐CD62L (1:50, from Miltenyi Biotec, Bergisch Gladbach, Germany), PE‐conjugated anti‐PD‐L1 (1:10, from Biolegend, CA, USA), and PE‐conjugated anti‐CD16 (1:50, from Miltenyi Biotec, Bergisch Gladbach, Germany). The cells were acquired with a MACS Quant Analyzer 10 (Miltenyi Biotec, Bergisch Gladbach, Germany) and analyzed with FlowJo v.10 software. Doublets and debris (identified according to forward and side scatter (SSC) properties), dead cells (identified with the Zombie Violet Fixable Viability Kit; BioLegend, San Diego, CA, USA), and eosinophils (identified using CCR3^+^ exclusion gating) were excluded from the analysis. The complete representations of the gating strategies used for PMNs and monocytes are depicted in Figures 1A–H and 2A–F, respectively.

Figure 1Representative flow cytometric panels were gated on live single cells and show forward scatter (FSC) and side scatter (SSC) images of purified PMNs (A, B). Since VioBlue‐positive cells included both dead cells and CCR3^+^ cells (eosinophils), both cell populations were excluded using a negative gate (C). PMNs were further identified as CD66b^+^ CD11b^+^ cells (D). Representative flow cytometric panels illustrating scatter plots of CD16^+^ CD62L^–^ cells in MPs (E) and HCs (G). Representative histograms illustrating PD‐L1^+^ PMNs in MPs (F) and HCs (H). Freshly isolated PMNs from the peripheral blood of MPs (black dots) and HCs (white dots) were stained for the neutrophil activation markers CD16 and CD62L (I) and PD‐L1 (J) at baseline and during follow‐up and then subjected to cytofluorimetric analysis. The results are expressed as mean ± SEM; Student’s *t*‐test or the Mann‒Whitney *U* test was used according to the parametric or nonparametric distribution of the variables.  ^∗∗∗∗^ *p* < 0.001. ROC curve analysis of serum levels of CD16^+^ CD62L^–^ cells (K) and PD‐L1^+^ PMNs (L) to evaluate the accuracy of cell frequencies as diagnostic biomarkers for MP. Area under the curve (AUC) = 0.66 and 0.8, respectively; cut‐off values = 7.5 and 50.6, respectively; sensitivity = 0.85 (CI 95% 0.66–0.97) and 0.8 (CI 95% 0.62–0.94), respectively; specificity = 0.54 (CI 95% 0.35–0.72) and 0.75 (CI 95% 0.60–0.93), respectively.

**Figure:**
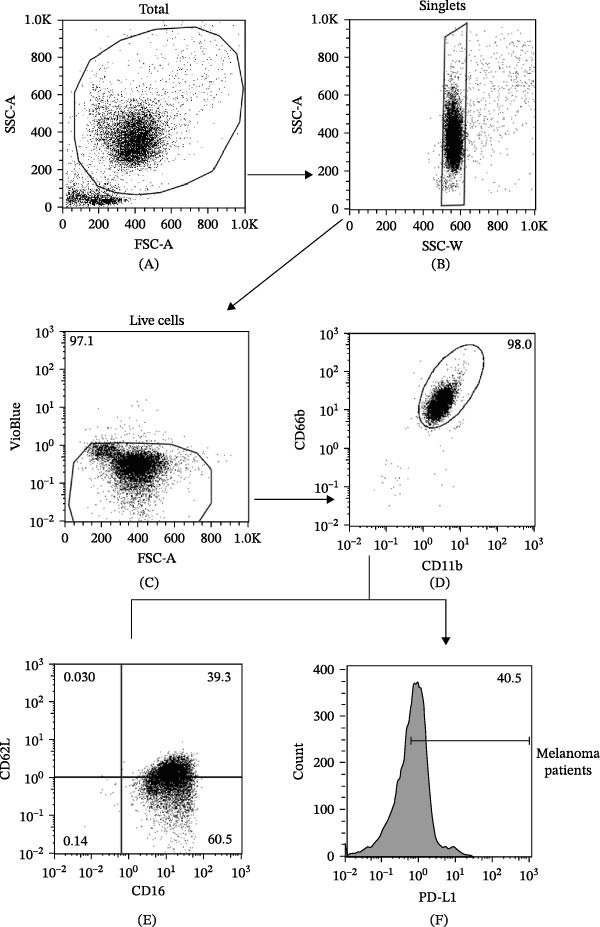

**Figure:**
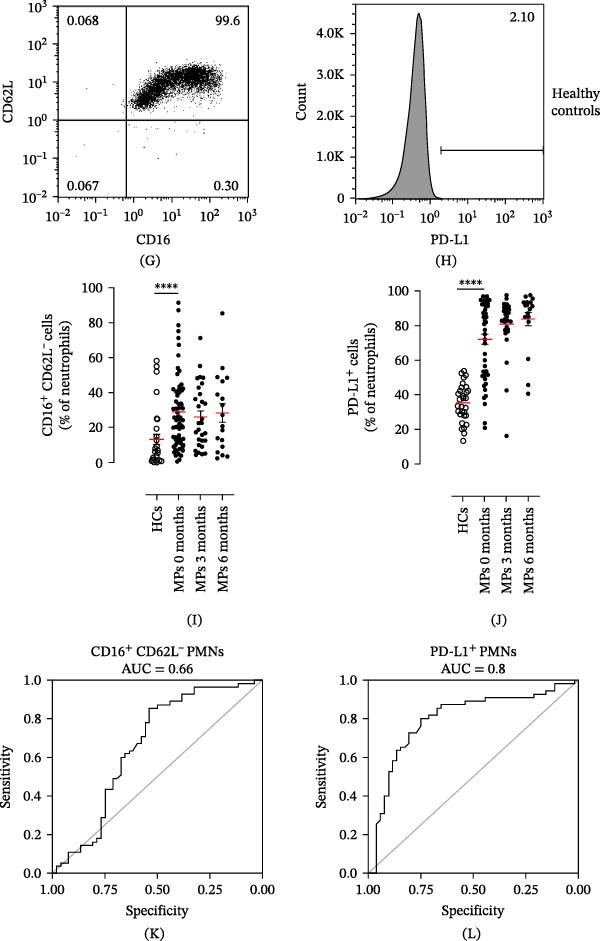

Figure 2Representative flow cytometric panels were gated on live single cells and show forward scatter (FSC) and side scatter (SSC) images of PBMCs (A, B). Dead cells were excluded from the analysis using a negative gate (C). Monocytes were further identified as CD14^+^ cells (D). Representative histograms illustrating PD‐L1^+^ monocytes from MPs (E) and HCs (F). CD14^+^ monocytes from the peripheral blood of MPs (black dots) and HCs (white dots) were stained for PD‐L1 (G) at baseline and during follow‐up and then subjected to cytofluorimetric analysis. The results are expressed as mean ± SEM; Student’s *t*‐test or the Mann‒Whitney *U* test was used to assess the parametric or nonparametric distribution of the variables.  ^∗∗∗∗^ *p* < 0.001. ROC curve analysis of serum levels of CD14^+^ monocytes (H) to evaluate the accuracy of cell frequencies as diagnostic biomarkers for MP. Area under the curve (AUC) = 0.71; cut‐off value = 63.0; sensitivity = 0.73 (CI 95% 0.56–0.87); specificity = 0.69 (CI 95% 0.54–0.87).

**Figure:**
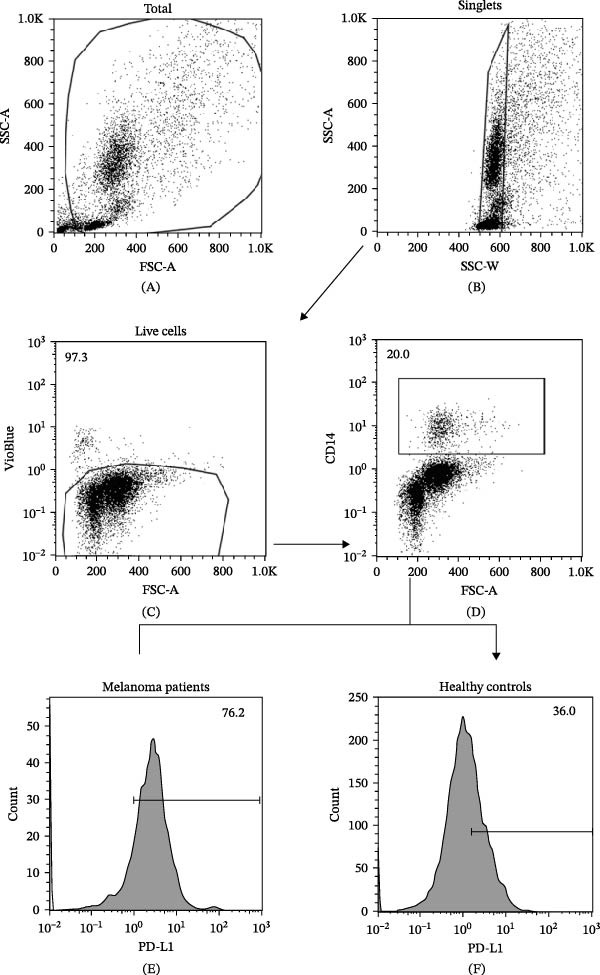

**Figure:**
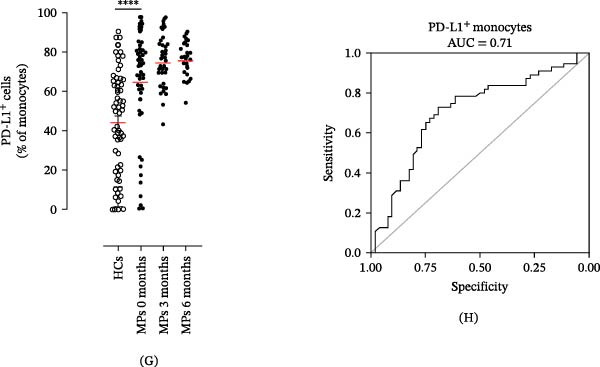

### 2.3. Quantification of Neutrophil‐Related Mediators

The concentrations of matrix metalloproteinase 9 (MMP‐9), myeloperoxidase (MPO), C‐X‐C motif chemokine ligand 8 (CXCL8)/interleukin‐8 (IL‐8), and granulocyte–macrophage colony‐stimulating factor (GM‐CSF) in the plasma of the MPs and HCs were determined by using commercially available enzyme‐linked immunosorbent assay (ELISA) kits (R&D Systems, Minneapolis, MN, USA). The absorbance of the samples was measured at 450 nm via a microplate reader (Tecan, Grödig, Austria). The ELISA detection ranges were 31.2–2000 pg/mL (for MMP‐9 and CXCL8/IL‐8), 62.5–4000 pg/mL (for MPO), and 15.6–1000 pg/mL (for GM‐CSF).

### 2.4. Plasma NET Biomarker Detection

Plasma MPO‒DNA complex levels of the MPs and HCs were measured as previously described [28]. Briefly, MaxiSorp 96‐well microplates were coated with a mouse monoclonal antihuman MPO antibody (5 mg/mL; Bio‐Rad, Hercules, CA, USA) diluted in PBS, incubated overnight at +4°C, and then blocked with 1% BSA in PBS. The samples and the anti‐DNA‐peroxidase (POD) antibody from the Cell Death Detection ELISA Kit (Roche, Basel, Switzerland) were added to the wells (120 min and +22°C). After incubation, the substrate solution from the Cell Death Detection ELISA Kit was added, and the absorbance was measured at 405 nm with a microplate reader (Tecan, Grӧdig, Austria). The concentration of citrullinated histone H3 (CitH3), a specific NET biomarker [35], was measured in the plasma of the MPs and HCs by an ELISA kit developed by Cayman Chemicals (Ann Arbor, MI, USA) according to the manufacturer’s instructions. The absorbance of CitH3 was determined at 450 nm. The ELISA sensitivity range was 0.15–10 ng/mL.

### 2.5. Tumor‐CM Preparation, Cell Cultures, and PD‐L1 Expression

ATCC provided the human melanoma cell lines SKMEL and A375, which were cultured and grown in RPMI 1640 supplemented with 10% heat‐inactivated fetal calf serum (FCS) (endotoxin level < 0.1 EU/mL), 50 U/mL penicillin/streptomycin, and 2 mM L‐glutamine (Euroclone, Milan, Italy) at 37°C in a humidified environment with 5% CO_2_ and 95% air. Adult lightly pigmented human epidermal melanocytes (HEMa‐LP) were cultured and maintained according to the manufacturer’s instructions (ThermoFisher, Waltham, MA, USA). Cells were cultured at a confluence of 10%–20% in tissue culture plates. When the cells reached 85%–90% confluence, fresh serum‐free media was added to the cell culture. After 24 h, the CM was collected, filtered through a 0.20‐µm pore size filter, and stored at −20°C [36] until use. All the cell lines were routinely tested for *Mycoplasma* using polymerase chain reaction (PCR) (Merk, Darmstadt, Germany). Neutrophils were purified from the peripheral blood of HC and stimulated with A375‐CM or control medium for 18 h. PMNs were stained (20 min and +4°C) in PBS containing 1% FBS with the following antibodies: APC‐conjugated anti‐CD66b (1:50, from Miltenyi Biotec, Bergisch Gladbach, Germany), PerCP‐conjugated anti‐CD11b (1:50, from Miltenyi Biotec, Bergisch Gladbach, Germany), VioBlue‐conjugated anti‐CD193 (1:10, from Miltenyi Biotec, Bergisch Gladbach, Germany), and PE‐conjugated anti‐PD‐L1 (1:10, from Biolegend, CA, USA). The cells were acquired with a MACS Quant Analyzer 10 (Miltenyi Biotec, Bergisch Gladbach, Germany) and analyzed with FlowJo v.10 software. Doublets and debris (identified according to forward and SSC properties), dead cells (identified with the Zombie Violet Fixable Viability Kit; BioLegend, San Diego, CA, USA), and eosinophils (identified using CCR3+ exclusion gating) were excluded from the analysis.

### 2.6. Lymphocyte Proliferation Assay

Neutrophils were suspended in RPMI supplemented with 5% FCS to a concentration of 1 × 10^6^/mL and stimulated with A375 CM or control medium for 18 h (37°C and 5% CO_2_). Lymphocytes of the same donor were highly purified with the Pan T Cell Isolation Kit according to the manufacturer’s instructions (Miltenyi Biotec, Bergisch Gladbach, Germany). Purified peripheral CD4^+^ T cells were labeled with CellTrace Violet Cell Proliferation Kit (Invitrogen, Thermo Fisher Scientific, Waltham, MA, USA). After 18‐h incubation, PMNs were washed twice in PBS, resuspended in RPMI supplemented with 5% FBS, and added in various ratios to the T cells. Proliferation was stimulated with anti‐CD3/CD28 (1 mg/mL) (Thermo Fisher Scientific, Waltham, MA, USA) and measured by flow cytometry after 3 days [37]. Specific blocking antibodies (anti‐PD‐L1, clone 29E.2A3, BioLegend, San Diego, USA) or the corresponding control isotype (R&D System, Minneapolis, MN, USA) were added to cell cultures at a concentration of 20 μg/mL at the time of stimulation [38]. Cells were incubated for 3 days, harvested, stained with anti‐CD4 antibodies (BD Biosciences, San Jose, CA, USA), acquired with a MACS Quant Analyzer 10 (Miltenyi Biotec, Bergisch Gladbach, Germany), and analyzed with FlowJo v.10 software.

### 2.7. Statistical Analysis

Statistical analyses were performed using GraphPad Prism 8 (GraphPad Software, La Jolla, CA, USA). The results are presented as mean ± SEM when quantitative variables were considered. Data normality was assessed via the D’Agostino and Pearson normality test. If the data were normally distributed at the 0.05 significance level, parametric tests were applied. For non‐normally distributed data, nonparametric tests were used. Depending on the parametric or nonparametric distribution of the variables, differences in cell subset frequencies between patients and controls or between patient subgroups were evaluated with Student’s *t*‐test or the Mann‒Whitney *U* test. A *p*‐value ≤0.05 was considered to indicate significance.

## 3. Results

### 3.1. Demographic and Clinical Characteristics of Stage III MPs

This study included 64 patients with stage III melanoma (MPs). The baseline clinicopathological characteristics of all patients are summarized in Table 1. The median age at diagnosis was 59 years; 44 patients (68.8%) were male, and 20 (31.3%) were female. Forty patients (62.5%) had melanoma harboring a *BRAF* mutation, and 24 (37.5%) had wild‐type *BRAF*. Regarding lymph node involvement, the MPs were distributed as follows: 5/65 (7.8%) had N0, 41/65 (64.1%) had N1, 5/65 (7.8%) had N2, and 2/65 (3.1%) had N3. MPs were divided into two subgroups according to the therapy: 42 received an IT, specifically anti‐PD‐1 therapy (nivolumab), and 22 received TT, specifically BRAF/MEK inhibitors (i.e., either dabrafenib or trametinib). These patients were further subdivided into the following two groups per their treatment response: “no evidence of disease” (NED—no clinical or radiographic evidence of disease recurrence) and “progressive disease” (PD—clinical or radiographic evidence of disease recurrence), both documented within 6 months from the beginning of therapy. Among the 42 MPs who received anti‐PD‐1 therapy, 20/42 (47.6%) experienced PD, and 22/42 (52.4%) had NED. In the BRAF/MEK subgroup, which included 22 MPs, 9/22 (40.9%) experienced PD, and 13/22 (59.1%) had NED.

### 3.2. Frequencies of CD16^+^ CD62L^–^ and PD‐L1^+^ PMNs and PD‐L1^+^ Monocytes in Stage III MPs

We prospectively investigated the frequencies of peripheral blood PMNs and monocytes via flow cytometry in all MPs participating in our study. To examine the activation state of PMNs, we measured CD16 and CD62L (L‐selectin) expression [31, 36, 39, 40]. Neutrophils express CD62L at rest; however, this expression quickly decreases (i.e., shedding) upon activation. Most CD16^bright^/CD62L^dim^ cells are neutrophils with hypersegmented nuclei, indicating an activated state [41]. Compared with HCs, stage III MPs presented increased percentages of activated CD16^+^ CD62L^–^ PMNs (Figure 1I). We then investigated the frequencies of peripheral blood PMNs and monocytes positive for PD‐L1 (PD‐L1^+^ PMNs and monocytes) via flow cytometry. The frequency of PD‐L1^+^ PMNs was higher in stage III MPs than in HCs (Figure 1J). No differences were found in CD16^+^ CD62L^–^ and PD‐L1^+^ PMN frequency over time during therapy (Figure 1I,J). Similar results were obtained for monocytes (Figure 2G). Importantly, receiver operating characteristic (ROC) curve analysis demonstrated that levels of blood circulating CD16^+^ CD62L^–^ PMNs, PD‐L1^+^ PMNs, and monocytes could discriminate MPs from HCs with high specificity and sensitivity (Figures 1K,L and 2H). Specifically, ROC curve analysis for CD16^+^ CD62L^–^ PMNs, PD‐L1^+^ PMNs, and monocytes showed cut‐off values of 7.5, 50.6, and 63.0, respectively, with sensitivity and specificity values of 0.85 and 0.54 for CD16^+^ CD62L^–^ PMNs, 0.80 and 0.75 for PD‐L1^+^ PMNs, and 0.73 and 0.69 for PD‐L1^+^ monocytes.

Collectively, our data revealed that peripheral blood PMNs from MPs were activated (CD16^+^ CD62L^–^) and displayed a greater percentage of PD‐L1 expression than did PMNs from HCs. In addition, MP peripheral blood monocytes presented higher frequencies of PD‐L1 than did those from HCs.

### 3.3. Frequencies of CD16^+^ CD62L^–^ and PD‐L1^+^ PMNs and PD‐L1^+^ Monocytes According to Patient Response to Therapy

We then assessed the frequencies of CD16^+^ CD62L^–^ PMNs, PD‐L1^+^ PMNs, and PD‐L1^+^ monocytes in the 64 patients with stage III melanoma, divided into subgroups according to therapy (anti‐PD‐1 and BRAF/MEK inhibitors) and considering the therapeutic response. In the group of patients receiving anti‐PD‐1 therapy, the basal percentages of CD16^+^ CD62L^–^ PMNs were greater in the PD subgroup than in the NED subgroup (Figure 3A). Moreover, the percentages of PD‐L1^+^ PMNs were also greater in the PD subgroup than in the NED subgroup (Figure 3B). Similar results were obtained for the basal levels of PD‐L1^+^ monocytes (Figure 3C). In patients treated with BRAF/MEK inhibitors, no difference in CD16^+^ CD62L^–^ PMN frequency at baseline was found between the PD and NED patient subgroups (Figure 3D). Similar results were found when we investigated the levels of PD‐L1^+^ PMNs and monocytes (Figure 3E,F). These results indicate that only within the IT subgroup, the basal levels of CD16^+^ CD62L^–^ and PD‐L1^+^ PMNs and PD‐L1^+^ monocytes increased in patients who progressed (PD subgroup) versus those who did not (NED subgroup). In contrast, within the TT subgroup, no differences in CD16^+^ CD62L^–^ and PD‐L1^+^ cell numbers were found between the PD and NED subgroups. These results suggest a differential predictive role of PMNs and monocytes in patients receiving anti‐PD‐1 IT compared with patients receiving anti‐BRAF/MEK TT.

Figure 3PMNs and monocytes from the peripheral blood of MPs (black dots). Freshly isolated PMNs from the peripheral blood of patients treated with anti‐PD‐1 therapy (A–C) or BRAF/MEK inhibitors (D–F) were stained for the activation markers CD16 and CD62L (A, D) or PD‐L1 (B, E), which were distributed into subgroups according to therapeutic response (PD, progressive disease; NED, no evidence of disease). CD14^+^ monocytes from the peripheral blood of MPs treated with anti‐PD‐1 therapy (C) or with BRAF/MEK inhibitors (F) were stained for PD‐L1 (C, F) and distributed into subgroups according to therapeutic response (PD, progressive disease; NED, no evidence of disease). The results are expressed as the mean ± SEM; Student’s *t*‐test or the Mann‒Whitney *U* test was used to assess the parametric or nonparametric distribution of the variables.  ^∗^ *p* < 0.05;  ^∗∗^ *p* < 0.01.

**Figure:**
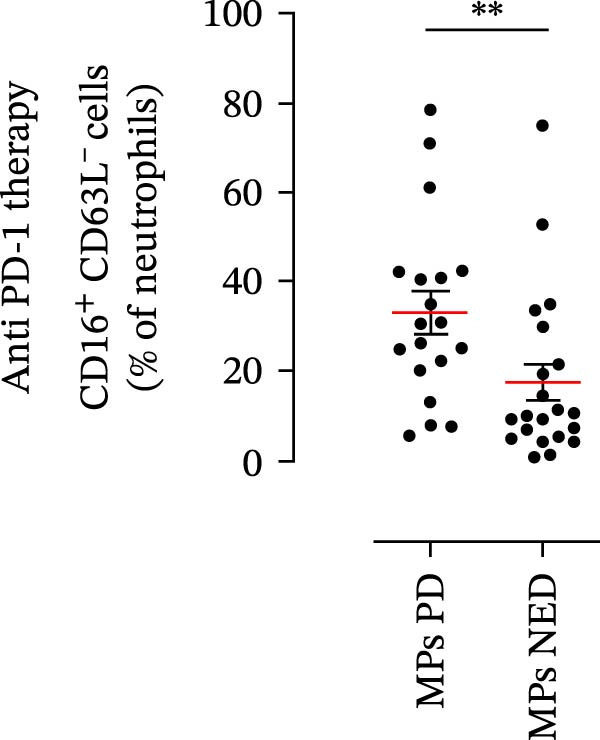
(A)

**Figure:**
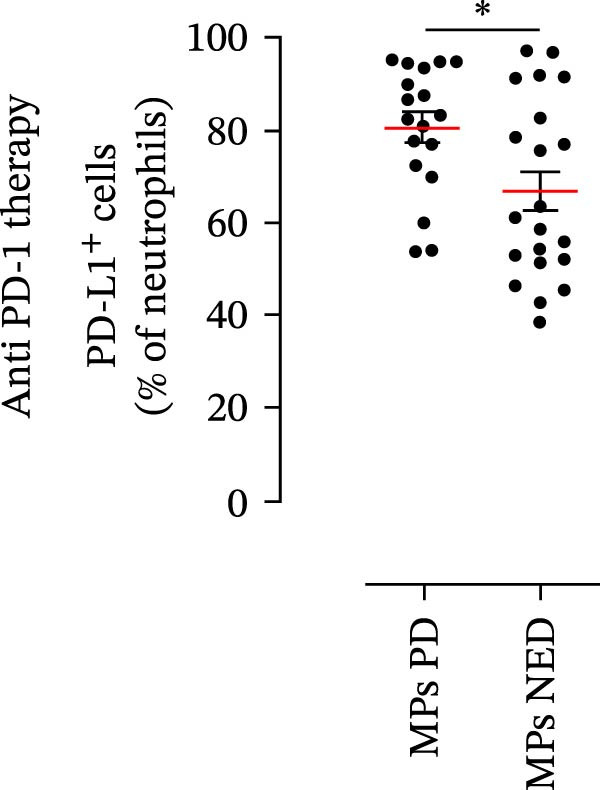
(B)

**Figure:**
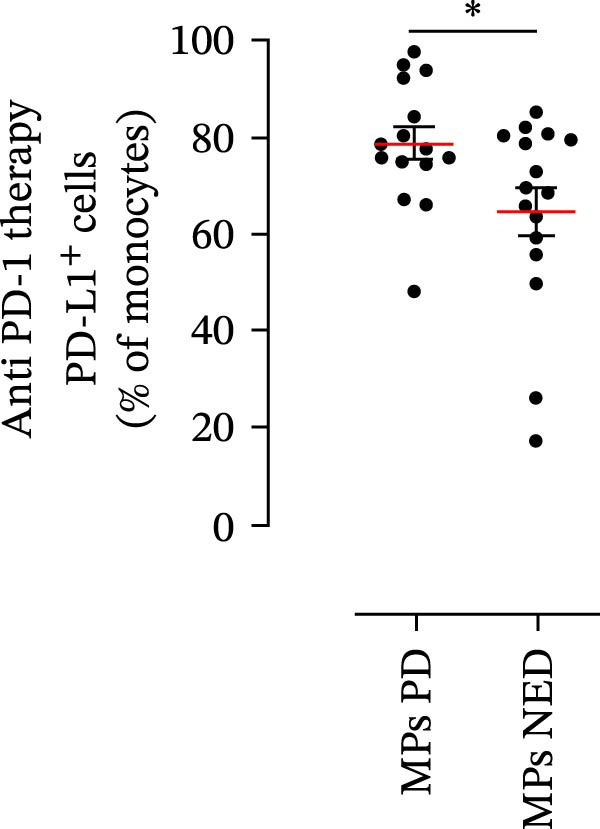
(C)

**Figure:**
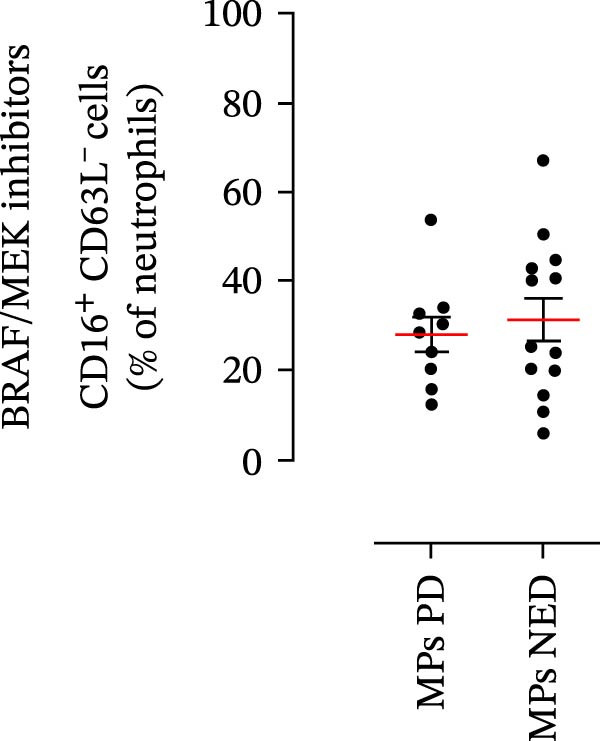
(D)

**Figure:**
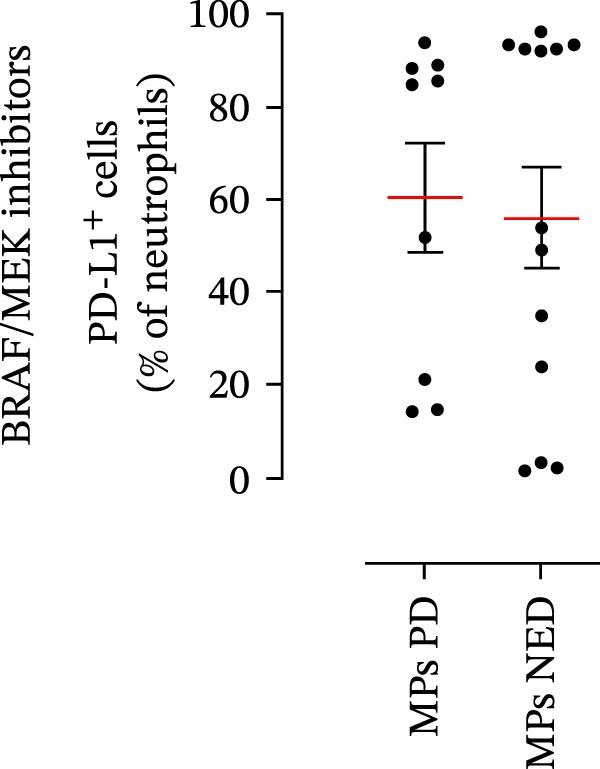
(E)

**Figure:**
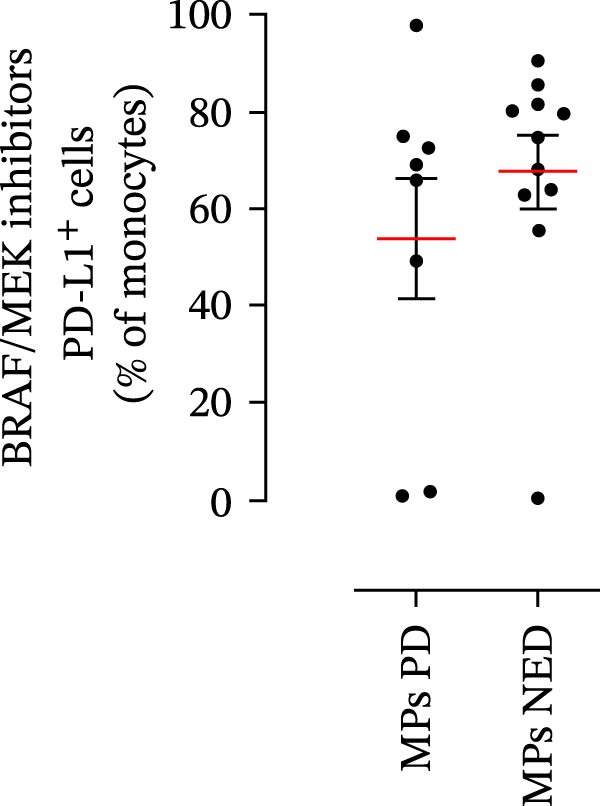
(F)

## 4. The CM of Stimulated Melanoma‐Derived Neutrophils Suppresses Lymphocyte Proliferation in a PD‐L1–Dependent Manner

PMNs were purified from the peripheral blood of HC, stimulated with the CM of melanoma cell lines SKMEL and A375 as well as of HEMa‐LP as primary cells or control medium for 18 h at 37°C, and then subjected to flow cytometry to evaluate the expression levels of PD‐L1. A375‐CM induced the overexpression of PD‐L1 on human PMNs after 18‐h incubation, compared to control medium and compared to the primary cell line HEMa‐CM (Figure 4A,B). These in vitro results support the hypothesis that our ex vivo observations relate to the capability of advanced melanoma cells to induce PD‐L1 expression on neutrophils in the patient.

Figure 4Neutrophils were cultured within HEMa, SKMEL, A375 cell line CMs, or CTRL medium for 18 h at 37°C. At the end of incubation, neutrophils were stained for PD‐L1 and subjected to cytofluorimetric analysis. Results were expressed as percentage of positive cells (A) or mean fluorescence intensity (B) gated on neutrophils (mean ± SEM of five independent experiments). Neutrophils were stimulated for 18 h at 37°C with A375‐CM or control medium, and inhibition of CD3/CD28‐induced CD4^+^ T cell proliferation was measured after 3 days, at different T cell‐PMN ratios, in the presence of anti‐PD‐L1 blocking antibody (C). Results were normalized for the corresponding isotype control and for the control medium and expressed as mean ± SEM; Student’s *t*‐test or Mann–Whitney *U* test according to the parametric or nonparametric distribution of variables.

**Figure:**
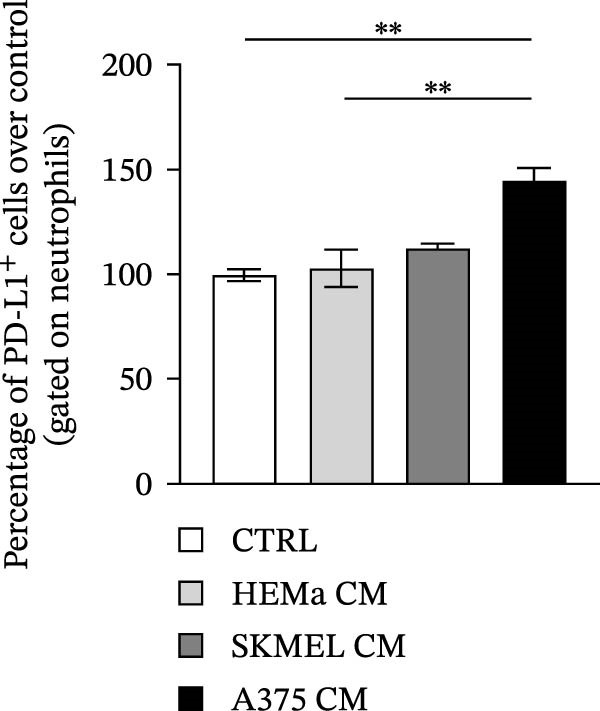
(A)

**Figure:**
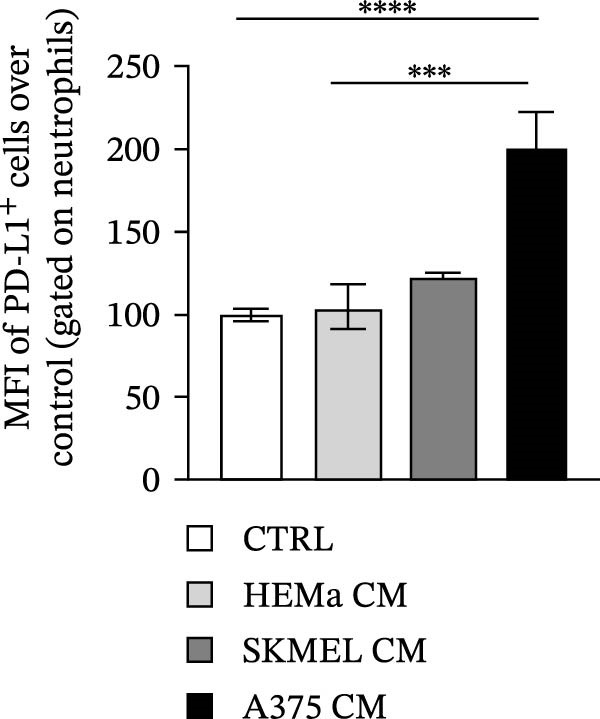
(B)

**Figure:**
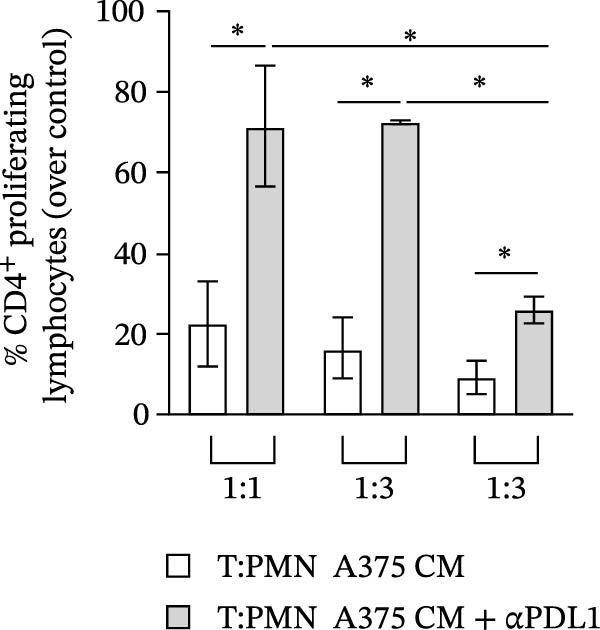
(C)

Since A375 CM was able to upregulate PD‐L1 expression on human PMNs, we investigated the capacity of PMNs stimulated with A375‐derived CM to suppress lymphocyte proliferation. Control medium–stimulated neutrophils showed modest suppression of CD3/CD28‐induced lymphocyte proliferation, whereas A375‐CM–stimulated neutrophils showed a robust (up to 70%) inhibition of CD4^+^ T cell proliferation (Figure 4C). To evaluate whether neutrophil‐mediated T cell suppression was dependent on PD‐L1, this protein was blocked, which hampered, at least in part, the PD‐L1^+^ PMN–induced lymphocyte suppression (Figure 4C).

### 4.1. Plasma Levels of Neutrophil‐Related Mediators and NET Biomarkers in Stage III MPs and According to Patient Response to Therapy

Human neutrophils produce and release several proinflammatory mediators and cytokines [19, 42]. The plasma levels of the neutrophil‐related mediators MMP‐9, MPO, CXCL8/IL‐8, and GM‐CSF (Figure 5A) were greater in stage III MPs than in HCs. The plasma concentrations of MPO–DNA complexes and CitH3, two specific biomarkers for NET identification, were also greater in MPs than in HCs (Figures 4F and 5E). These results support the hypothesis that neutrophils are activated in stage III MPs.

Figure 5Plasma concentrations of MMP‐9 (A), MPO (B), CXCL8/IL‐8 (C), and GM‐CSF (D) in MPs (black dots) and healthy controls (HCs, white dots) were measured by ELISA. Plasma levels of MPO–DNA complexes (E) and citrullinated histone H3 (CitH3) (F) in MPs (black dots) and healthy controls (HCs, white dots) were evaluated by ELISA or by citrullinated histone H3 (clone 11D3) ELISA kit (Cayman), respectively. The results were expressed as mean ± SEM; Student’s *t*‐test or Mann–Whitney *U* test according to the parametric or nonparametric distribution of variables.  ^∗^ *p* < 0.05;  ^∗∗^ *p* < 0.01;  ^∗∗∗∗^ *p* < 0.001.

**Figure:**
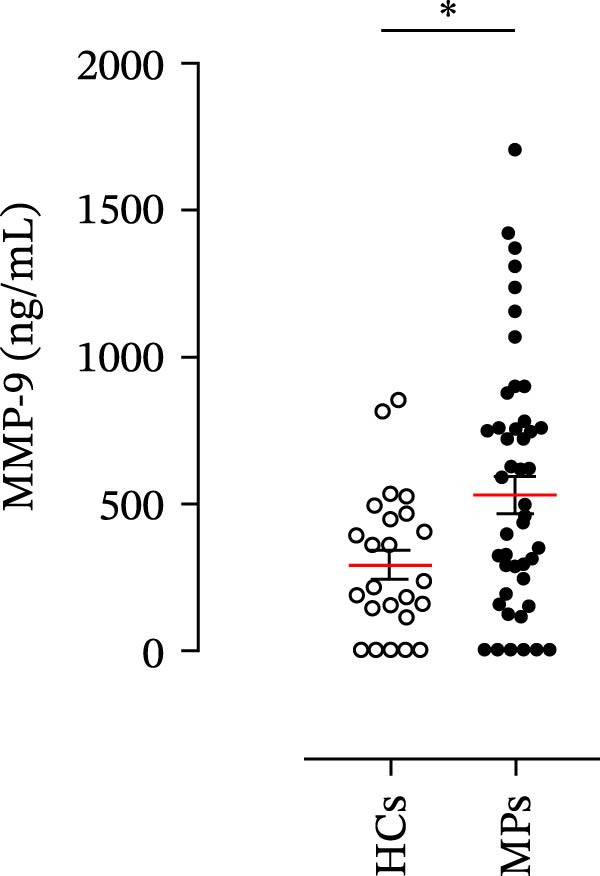
(A)

**Figure:**
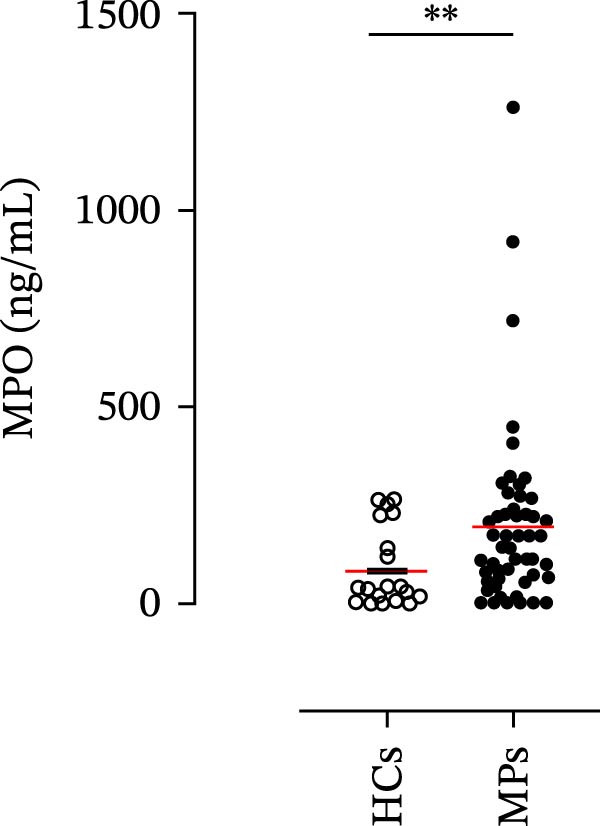
(B)

**Figure:**
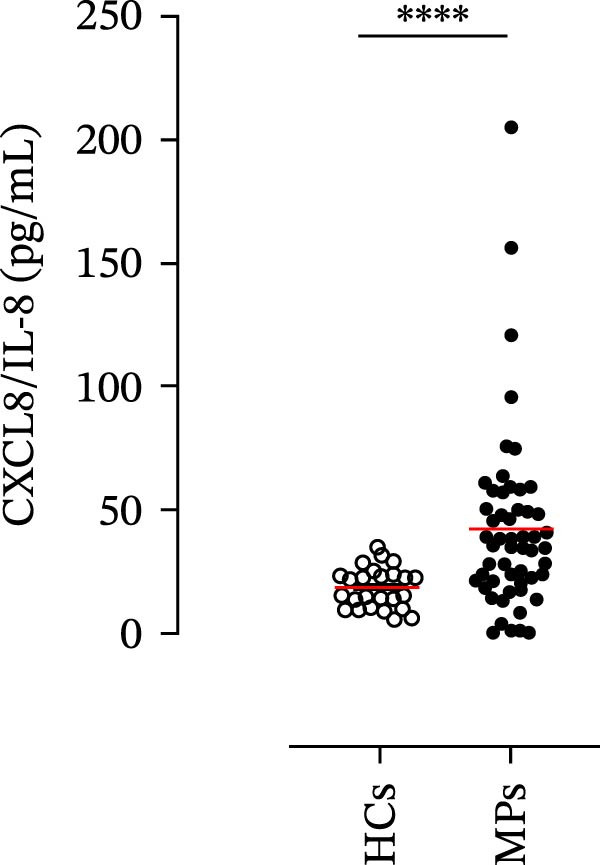
(C)

**Figure:**
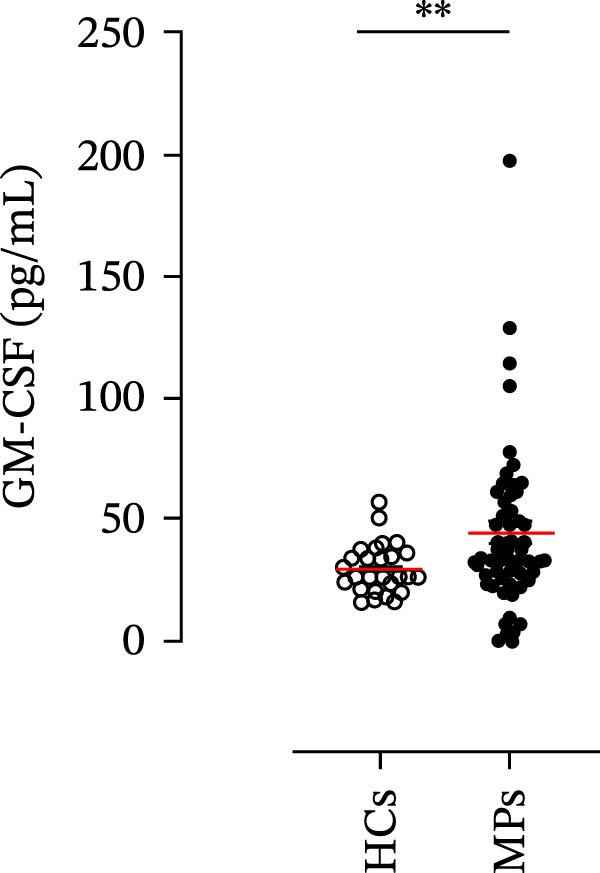
(D)

**Figure:**
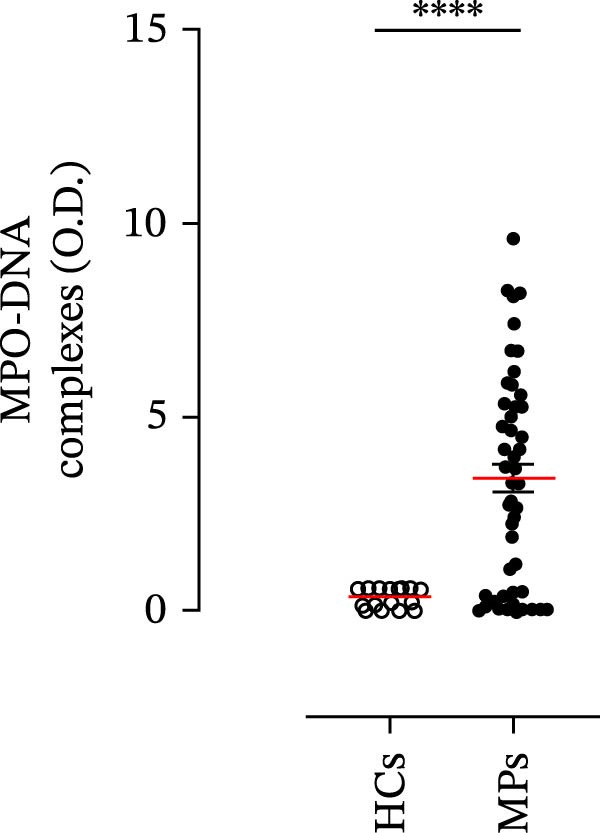
(E)

**Figure:**
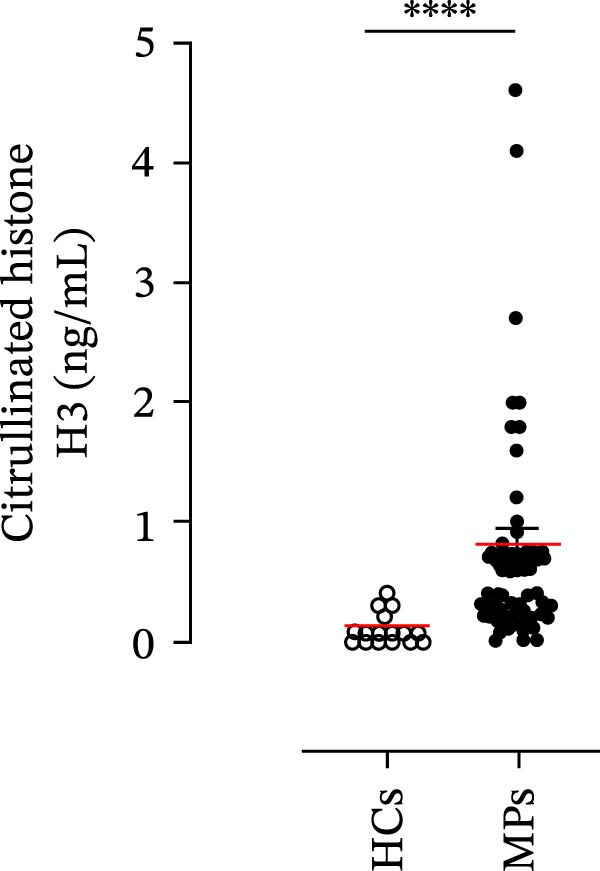
(F)

The plasma levels of neutrophil‐related mediators and NET biomarkers were then analyzed in the different patient subgroups according to the therapy (IT versus TT) and clinical response (PD or NED). Interestingly, in both the IT (Figure 6A–D) and TT (Figure 6E–H) patient subgroups, no differences were found in the circulating levels of MMP‐9 (Figures 5E and 6A), MPO (Figures 5F and 6B), CXCL8/IL‐8 (Figures 5G and 6C), or GM‐CSF (Figures 5H and 6D). Similarly, both in patients treated with anti‐PD‐1 therapy (Figures 6B and 7A) and in those treated with BRAF/MEK inhibitors (Figures 6D and 7C), the plasma concentrations of NET biomarkers (MPO–DNA complexes and CitH3) were not differentially distributed between PD and NED MPs (Figure 7A–D). Finally, no differences were found between the PD subgroup and the NED subgroup, regardless of the therapy used.

Figure 6Plasma concentrations of MMP‐9, MPO, CXCL8/IL‐8, and GM‐CSF in MPs (black dots) treated with anti‐PD‐1 therapy (A–D) or in MPs (black dots) treated with BRAF/MEK inhibitors (E–H) were measured via ELISA. The results were distributed into subgroups according to therapeutic response (PD, progressive disease; NED, no evidence of disease) and expressed as the mean ± SEM; Student’s *t*‐test or the Mann‒Whitney *U* test was used according to the parametric or nonparametric distribution of the variables.

**Figure:**
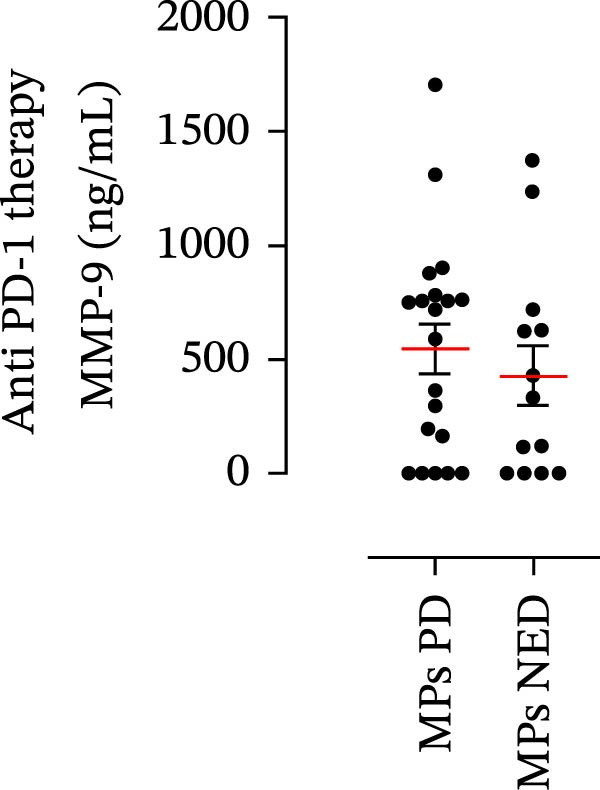
(A)

**Figure:**
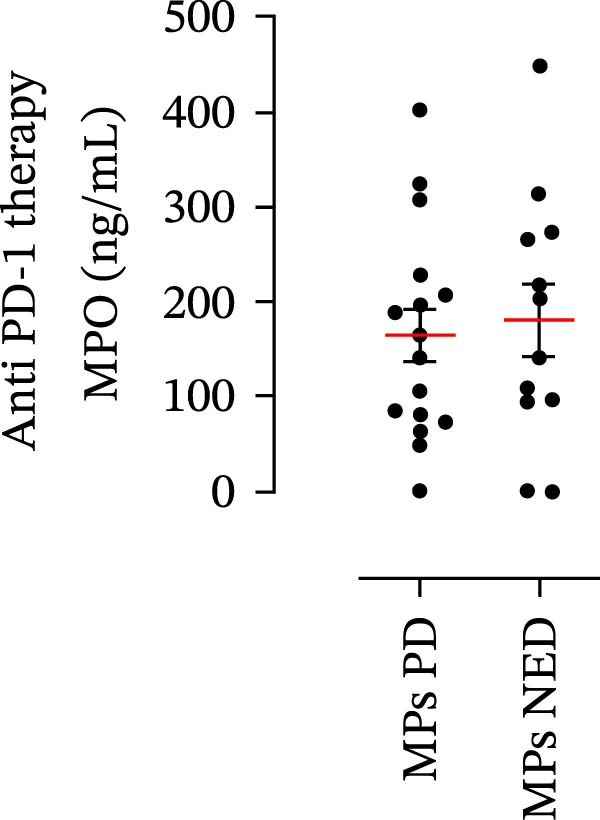
(B)

**Figure:**
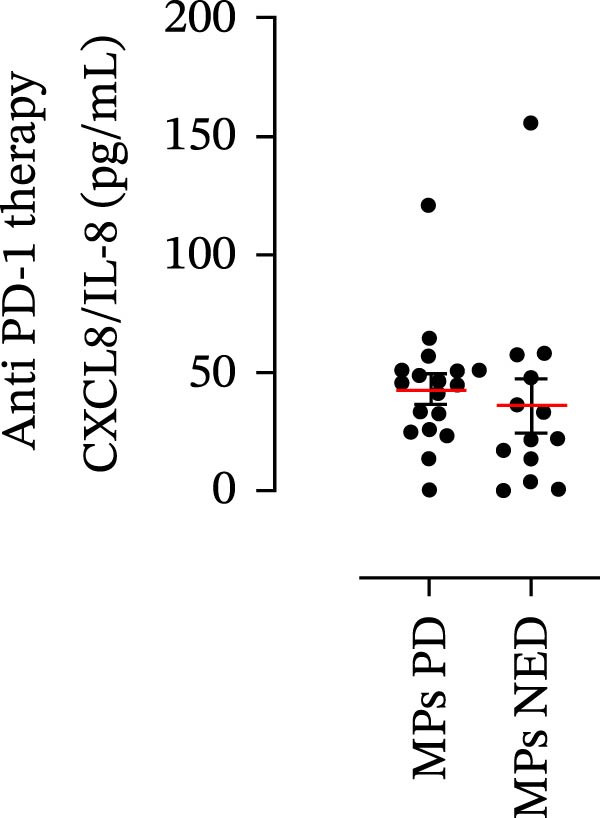
(C)

**Figure:**
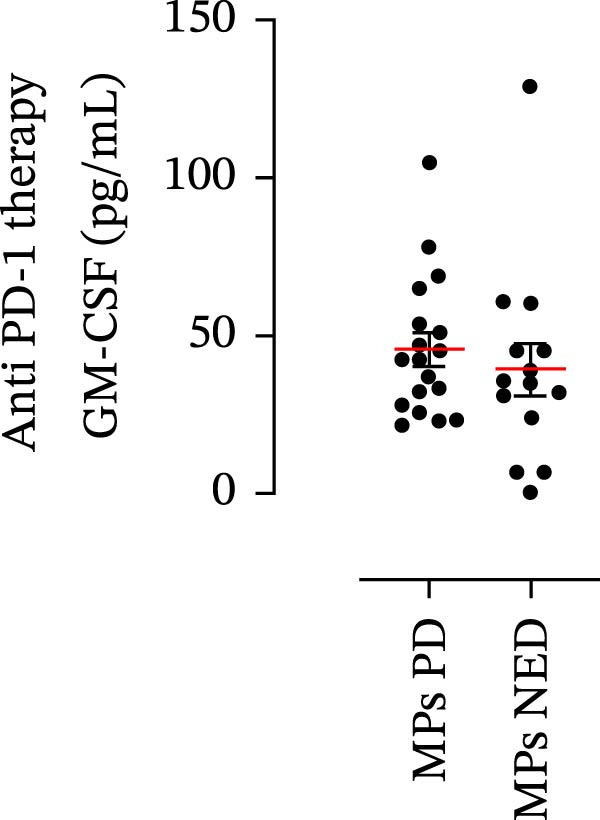
(D)

**Figure:**
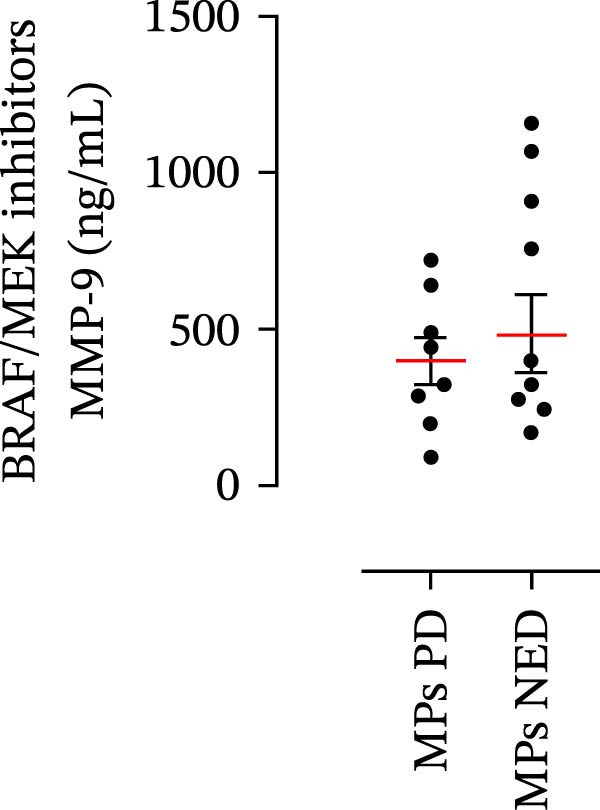
(E)

**Figure:**
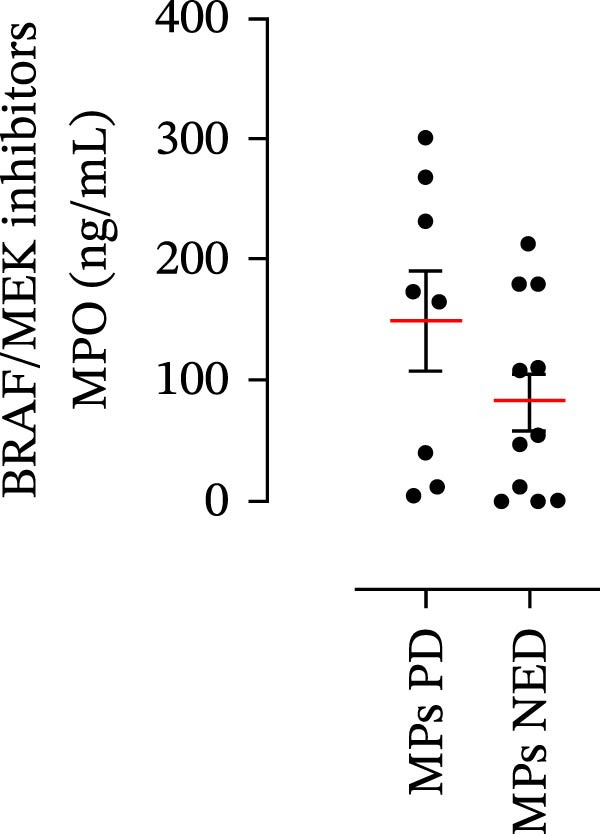
(F)

**Figure:**
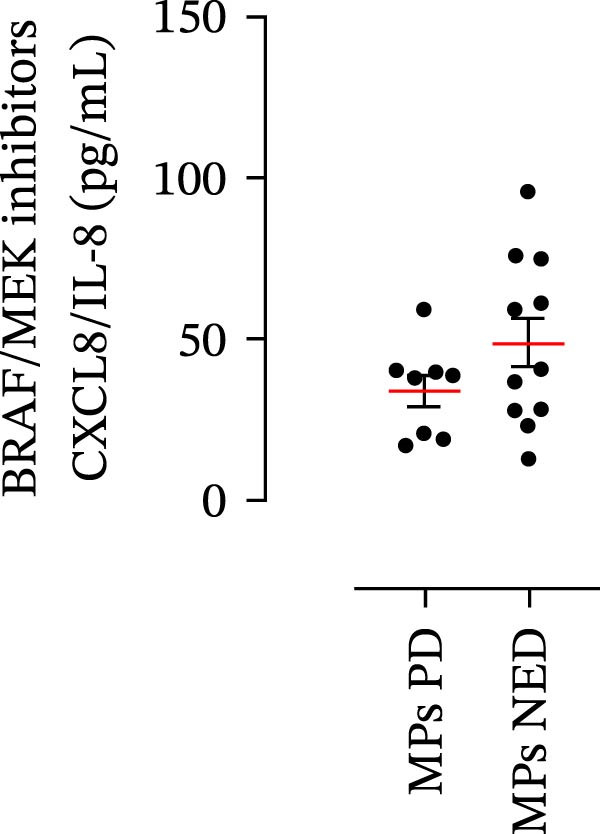
(G)

**Figure:**
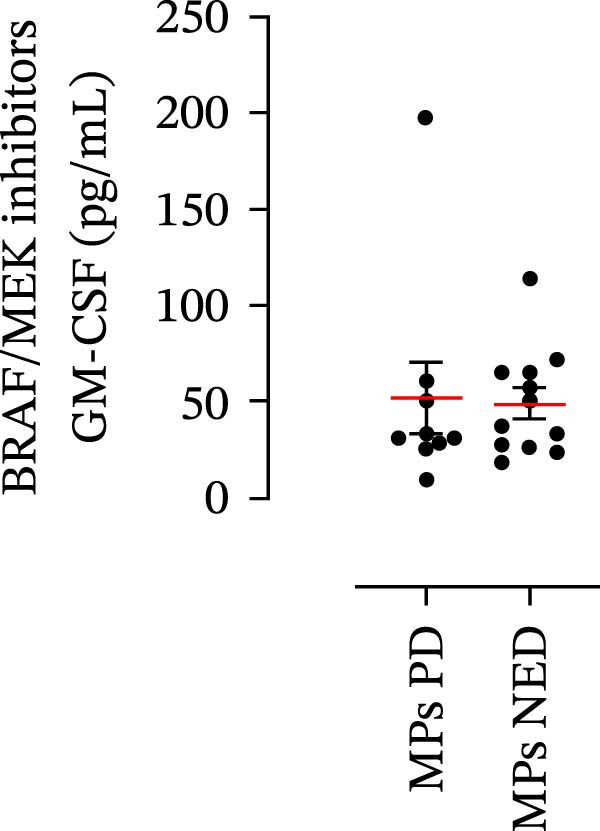
(H)

Figure 7The plasma levels of MPO‒DNA complexes and citrullinated histone H3 (CitH3) in MPs (black dots) treated with anti‐PD‐1 therapy (A, B) or in MPs (black dots) treated with BRAF/MEK inhibitors (C, D) were evaluated via ELISA or a citrullinated histone H3 (clone 11D3) ELISA kit (Cayman), respectively. The results were distributed into subgroups according to therapeutic response (PD, progressive disease; NED, no evidence of disease) and expressed as mean ± SEM; Student’s *t*‐test or the Mann‒Whitney *U* test was used according to the parametric or nonparametric distribution of the variables.

**Figure:**
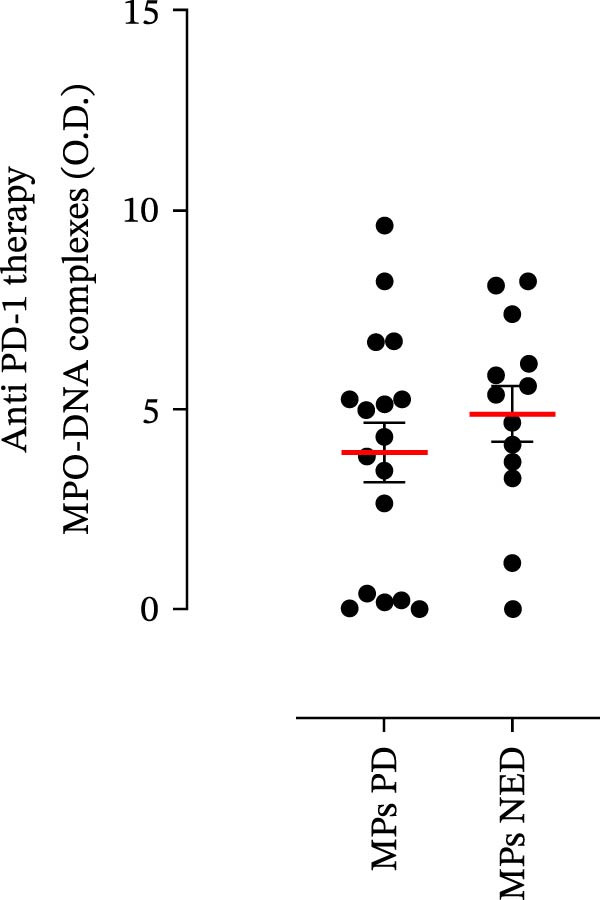
(A)

**Figure:**
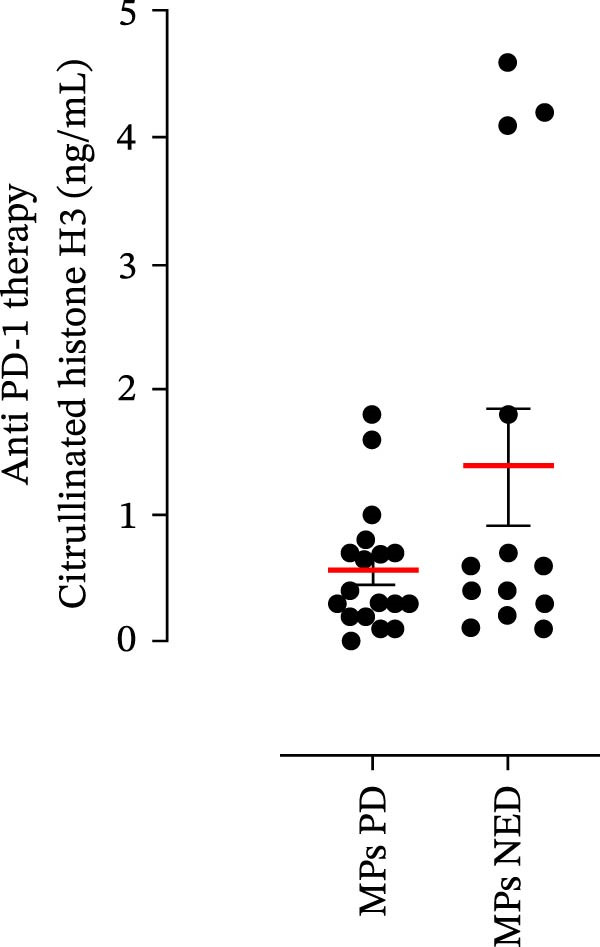
(B)

**Figure:**
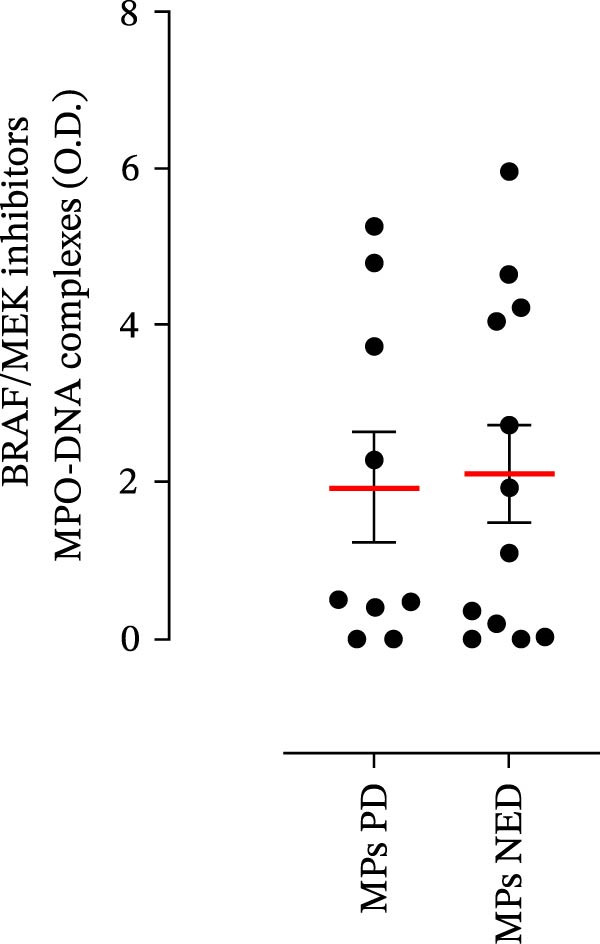
(C)

**Figure:**
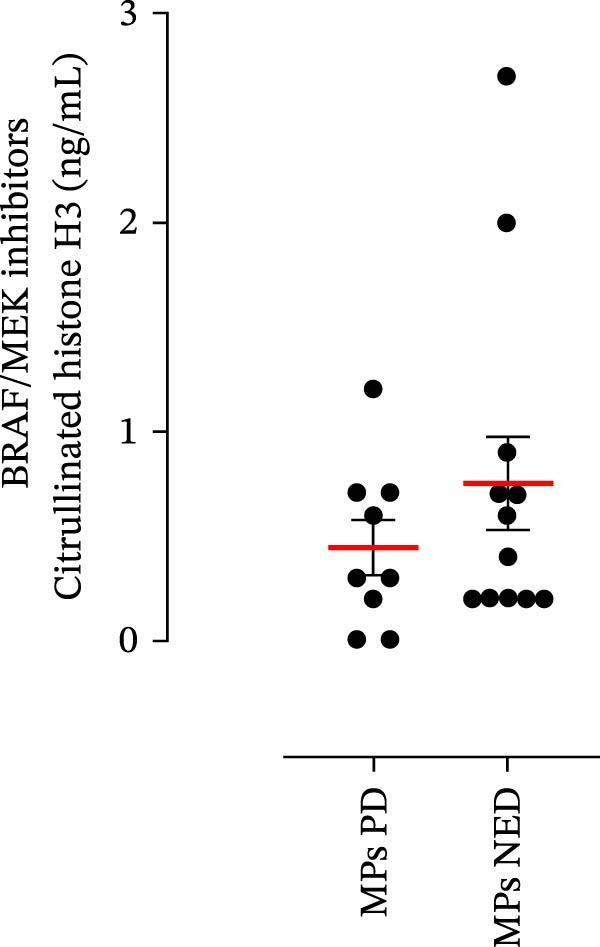
(D)

## 5. Discussion

The treatment of metastatic melanoma has been revolutionized by a wave of novel therapies, including BRAF/MEK inhibitors and first‐ and second‐generation immunotherapies (anti‐CTLA‐4 and anti‐PD‐1 checkpoint blockade) [43, 44]. As a result, the 5‐year relative survival rate for patients with distant‐stage melanoma has more than doubled, rising from 18% in 2009 to 38% in 2015 [45].

This study investigated the phenotype and functional role of circulating neutrophils and monocytes in patients with stage III melanoma receiving adjuvant IT or TT. By integrating clinical observations with functional in vitro experiments, we provide evidence that specific innate immune cell phenotypes are differentially associated with clinical outcomes depending on the therapeutic strategy. Specifically, we recruited 64 patients with stage III melanoma; of these patients, 42 were treated with anti‐PD‐1 therapy, and 22 were treated with BRAF/MEK inhibitors.

We first evaluated the frequencies of CD16^+^ CD62L^–^ and PD‐L1^+^ PMNs and PD‐L1^+^ monocytes in the peripheral blood of MPs before and during therapy. First, through flow cytometry, we measured the frequencies of CD16^+^ CD62L^–^ PMNs, which were greater in MPs than in HCs at the beginning of and during treatment. The CD16^+^ CD62L^–^ subset is a peculiar subgroup of PMNs that shows an immunosuppressive profile [46]. The percentages of these subset PMNs were increased in patients with different types of solid and hematological tumors [46, 47], fully reflecting the observations in our cohort. In addition, we found that stage III MPs had higher baseline frequencies of PD‐L1^+^ PMNs in the peripheral blood than did HCs, unaffected by IT. Importantly, ROC curve analysis demonstrated that circulating levels of CD16^+^ CD62L^–^ PMNs, PD‐L1^+^ PMNs, and monocytes could serve as a potential biomarker for distinguishing MP patients from healthy individuals (Figures 1K,L and 2H).

An increasing number of studies have emphasized the importance of PD‐L1^+^ neutrophils in cancer progression. In hepatocellular carcinoma (HCC), PD‐L1^+^ PMNs aggregate in peritumoral areas, suppressing T cell activation and proliferation [22]. Tumor‐derived GM‐CSF suppressed T cell immunity and aided in the progression of gastric cancer (GC) by inducing PD‐L1 expression on PMNs through the Jak‐Stat3 signaling pathway [23]. Patients with poorly differentiated HCC have considerably increased levels of PD‐L1^+^ PMNs in their peripheral blood, which independently predict poor prognosis [48]. In line with these findings, our results revealed an increase in the frequency of PD‐L1^+^ PMNs, suggesting potential tumor‐promoting effects of these cells in stage III MPs.

Recently, CD14^+^ cells from patients with different types of cancer have been found to express PD‐L1 [49–51]. Natural killer/T cell lymphoma (NKTCL) patients display a high percentage of peripheral blood PD‐L1^+^ monocytes and high frequencies of PD‐L1^+^ monocytes in tumor tissue [52]. Increased PD‐L1 and PD‐L2 expression on the monocytes of HCC patients is also associated with a poor prognosis [50]. Our results revealed that stage III MPs have higher levels of PD‐L1^+^ monocytes than HCs, both at the beginning of and during therapy, suggesting a potential protumor profile of monocytes in our cohort of patients, similar to that of neutrophils and consistent with previous reports [49, 50, 52].

Specifically, within the two patient subgroups (patients treated with anti‐PD‐1 therapy and those treated with BRAF/MEK inhibitors), the patients were further divided into two additional subgroups, namely, the PD and NED subgroups, according to their therapeutic response. Our results revealed a significant difference between the two therapeutic settings. Higher frequencies of CD16^+^ CD62L^–^ and PD‐L1^+^ PMNs and PD‐L1^+^ monocytes were found in the PD subgroup but only in the group treated with anti‐PD‐1 therapy. In contrast, no differences between the PD and NED subgroups were found among patients treated with TT. These findings suggest that innate immune cells can influence the effectiveness of PD‐1 blockade treatment. Moreover, these data demonstrate that the frequencies of activated PMNs, PD‐L1^+^ neutrophils, and monocytes are inversely associated with the clinical benefit of anti‐PD‐1 therapy, suggesting their potential as predictive biomarkers.

In subsequent experiments, we investigated the plasma levels of neutrophil‐related mediators. We detected higher plasma concentrations of neutrophil‐associated factors (MMP‐9, MPO, CXCL8/IL‐8, and GM‐CSF) in MPs than in HCs. Some of these mediators (i.e., MMP‐9 and GM‐CSF) are involved in melanoma progression [53, 54]. We also recently demonstrated the involvement and presence of these circulating neutrophil‐related mediators in stage IV MPs [31]. Furthermore, we investigated the circulating levels of NET biomarkers assessed by MPO‒DNA complexes and CitH3, two of the most reliable indicators of NET formation [28, 35]. Our results revealed that stage III MPs presented higher plasma levels of neutrophil‐related mediators and NET biomarkers than HCs. We then analyzed the differences in the plasma levels of these mediators between the two therapy patient groups, dividing them into PD and NED subgroups. Interestingly, unlike the results obtained with PMN and monocytes, no difference in the plasma levels of these neutrophil‐related mediators was found between the PD and NED subgroups in either patient subgroup (IT or TT). Similar results were found for the NET biomarkers. These results appear to contrast with the differences found in the frequencies of PD‐L1^+^ and CD16^+^ CD62L^–^ PMNs and PD‐L1^+^ monocytes. However, these apparently conflicting results further highlight the specificity of the role of the cells in predicting patient clinical response compared with soluble mediators in patients undergoing anti‐PD‐1 IT. The lack of association between circulating neutrophil‐derived mediators or NET biomarkers and clinical response, despite clear differences at the cellular level, suggests that cell‐intrinsic immune phenotypes may provide more informative insights than soluble mediators. This observation underscores the importance of immune cell profiling over systemic inflammatory markers when investigating mechanisms of resistance to immune checkpoint blockade.

Recently, combined single‐cell RNA sequencing and dataset analysis such as The Cancer Genome Atlas (TCGA) allowed the obtaining of neoplasm gene expression data that correlated with drug resistance predictions [55]. This system demonstrates high efficacy and can be applied to various cancer types. The findings indicate that integrating multiple approaches is likely to shape the future of oncology pharmacogenetics. By tailoring treatment to individual genetic profiles, this strategy could increase survival rates and potentially reduce both patient side effects and healthcare costs. Customizing anticancer drugs based on disease‐associated biomarkers and drug‐specific genetic variants may further enhance treatment outcomes.

Predictive markers for the response to IT and TT are needed, and successful therapy might depend on both innate and adaptive immunity [15]. The potential role of innate immune cells as predictive biomarkers in patients treated with anti‐PD‐1 therapy is currently under debate. Indeed, several studies have shown that high PD‐L1 expression in PMNs and monocytes is correlated with a poor response to anti‐PD‐1 therapy in patients with different cancers, including melanoma [30, 56]. We recently demonstrated the involvement of PD‐L1^+^ PMNs in patients with stage IV melanoma treated with anti‐PD‐1 IT [30]. In a nonclassical and intermediate monocyte subset, PD‐L1 expression was significantly increased in advanced MPs, who demonstrated shorter PFS times, and inversely correlated with OS rates [56]. Our data shed new light on the predictive role of innate immune cells in MPs. For the first time, we analyzed the phenotypes of circulating innate immune cells in patients treated with anti‐PD‐1 therapy and those treated with TT. Importantly, in stage III MPs, neutrophil and monocyte phenotypes were associated with better responses to ICI therapy; however, this effect was not observed in TT‐treated patients, highlighting the dual clinical significance of innate immune cells, depending on the different therapies administered.

This selectivity in the predictive power of the variables could be explained by the potential role of PD‐L1^+^ PMNs and PD‐L1^+^ monocytes in melanoma progression. Indeed, PD‐L1^+^ PMNs and PD‐L1^+^ monocytes could exert a direct impact on the ICI mechanism of action since the expression of PD‐L1 contributes to T cell inactivation. The resulting T cell suppression enables tumor cells to avoid immune surveillance and proliferate. Consequently, the increased percentages of PD‐L1^+^ PMNs and PD‐L1^+^ monocytes could reduce the ICI therapeutic effect, increasing melanoma progression despite treatment. On the other hand, the lack of predictive power for TT (targeting BRAF/MEK) could be explained by the direct effects of the BRAF/MEK inhibitors, beyond the immunological roles of innate immune cells.

From the mechanistic point of view, we investigated the ability of PD‐L1^+^ PMNs to induce T cell suppression. To this aim, we took advantage of an in vitro model. We first evaluated the ability of melanoma‐derived CM to induce PD‐L1 on normal PMNs. Exposure to melanoma‐derived CM increased the expression of PD‐L1 on human PMNs. We already demonstrated the multiple effects of melanoma‐derived CM on PMN functions such as stimulation of the respiratory burst, increased ex vivo survival, and NET release [31]. Here, we demonstrate that the A375‐CM induced PD‐L1 expression on normal PMNs, thus recapitulating the results obtained ex vivo in advanced MPs.

In addition, we found that A375‐CM–stimulated PMNs significantly suppressed lymphocyte proliferation, and this suppressive effect was dependent on increased expression of PD‐L1 on PMNs. Indeed, under steady‐state conditions, expression of PD‐L1 on PMNs is very low, and these PMNs show only a minor suppressive phenotype ex vivo. A375‐CM stimulation of PMNs resulted in a dose‐dependent suppressive activity. By blocking PD‐L1 on PMNs, we verified that suppression of lymphocyte proliferation was dependent on PD‐L1–PD‐1 signaling. To our knowledge, we are the first to identify an immunosuppressive effect of A375‐CM–induced PD‐L1^+^ PMNs.

We acknowledge that the limited sample size represents a limitation of this study and may have reduced the statistical power of subgroup analyses, particularly those aimed at formally assessing predictive performance. Nevertheless, the prospective design, the homogeneity of the cohort, and the consistent differences observed between treatment groups support the biological relevance of our findings. Moreover, we found a potential mechanism underlying the immunosuppressive function of melanoma “primed” PD‐L1^+^ PMNs in vivo. Larger multicenter studies with longer follow‐up will be required to validate these observations and assess their potential clinical utility.

## 6. Conclusions

Our study highlights a therapy‐dependent role of circulating neutrophils and monocytes in stage III melanoma. Elevated frequencies of activated and PD‐L1–expressing innate immune cells were associated with disease progression in patients receiving anti‐PD‐1 therapy but not in those treated with BRAF/MEK inhibitors. By integrating clinical data with functional evidence, these findings support a model in which innate immune cell–mediated immunosuppression may interfere with the efficacy of immune checkpoint blockade.

This work provides a rationale for further investigation of innate immune biomarkers in melanoma and supports the development of integrated immune profiling approaches to better understand mechanisms of resistance to IT.

**Nomenclature**
CitH3: Citrullinated histone H3
CTLA‐4: Cytotoxic T lymphocyte‐associated antigen 4
GM‐CSF: Granulocyte–macrophage colony‐stimulating factor
ICIs: Immune checkpoint inhibitors
IT: Immunotherapy
HCs: Healthy controls
MPs: Melanoma patients
MPO: Myeloperoxidase
MMP‐9: Matrix metalloproteinase 9
NED: No evidence of disease
NETs: Neutrophil extracellular traps
ORR: Overall response rate
OS: Overall survival
PD: Progressive disease
PD‐1: Programmed cell death protein 1
PD‐L1: PD‐1 ligand
PFS: Progression‐free survival
PMNs: Polymorphonuclear leukocytes
TME: Tumor microenvironment
TT: Targeted therapy.

## Author Contributions

Annagioia Ventrici, Luca Modestino, and Maria Rosaria Galdiero were responsible for conceptualization, formal analysis, writing – original draft preparation, and writing – reviewing and editing. Annagioia Ventrici, Ilaria Spatocco, Leonardo Cristinziano, and Francesco Caraglia assisted with the methodology. Leonardo Cristinziano, Carlo Gabriele Tocchetti, and Remo Poto helped with software. Maria Rosaria Galdiero carried out supervision, administered the project, and acquired the funding. Gilda Varricchi and Paola de Candia participated in validation. Luca Modestino, Annagioia Ventrici, and Teresa Troiani conducted the investigation. Teresa Troiani and Maria Rosaria Galdiero contributed to resources. Annagioia Ventrici, Ilaria Spatocco, and Luca Modestino curated the data. Remo Poto took part in the visualization. Paola de Candia and Maria Rosaria Galdiero did the supervision.

## Funding

This work was supported by AIRC under MFAG 2020 (Grant 25123) and partially by PRINPNRR2022 P202282S5M, PNRR‐MAD‐2022‐12376769, and PNRR‐TR1‐2023‐12377980. Open access publishing is facilitated by Universita degli Studi di Napoli Federico II as part of the Wiley–CRUI‐CARE agreement.

## Disclosure

All authors have read and agreed to the published version of the manuscript.

## Conflicts of Interest

The authors declare no conflicts of interest.

## Data Availability

The data presented in this study are available upon request from the corresponding author.
